# HE4 Tissue Expression as A Putative Prognostic Marker in Low-Risk/Low-Grade Endometrioid Endometrial Cancer: A Review

**DOI:** 10.3390/curroncol29110673

**Published:** 2022-11-10

**Authors:** Valerio Mais, Maria Luisa Fais, Michele Peiretti, Daniela Fanni, Elena Massa, Giulia Carboni, Giuseppina Fais, Giuseppe Deo, Stefano Angioni

**Affiliations:** 1 Department of Surgical Sciences, University of Cagliari Medical School, 09042 Cagliari, Italy; 2 Division of Gynecology and Obstetrics (AOU di Cagliari), Department of Surgical Sciences, University of Cagliari, 09042 Cagliari, Italy; 3 Division of Pathology (AOU di Cagliari), Department of Medical Sciences and Public Health, University of Cagliari, 09042 Cagliari, Italy; 4 Division of Oncology (AOU di Cagliari), Department of Medical Sciences and Public Health, University of Cagliari, 09042 Cagliari, Italy

**Keywords:** endometrial cancer, endometrial hyperplasia, cancer genome atlas, human epididymis protein 4, HE4, immunohistochemistry, mRNA, tissue expression

## Abstract

Low-grade stage I endometrioid endometrial carcinomas should have an excellent prognosis, but a small subset of these cancers can relapse. The search for putative immunohistochemical prognostic markers for relapse in low-risk/low-grade endometrioid endometrial cancers remains open. Among the candidate molecules that may implicate the roles of immunohistochemical risk markers, we focused our attention on human epididymis protein 4 (HE4) after a review of the literature. Few authors have devoted themselves to this topic, and none have found a correlation between the tissue expression of HE4 and the molecular classification of endometrial cancer. Five different variants of HE4 mRNA and multiple protein isoforms of HE4 were identified many years ago, but current HE4 assays only measure the total HE4 expression and do not distinguish the different proteins encoded by different mRNA variants. It is important to have an approach to distinguish specific variants in the future.

## 1. Introduction

In the last ten years, the classification of endometrial cancer has undergone numerous updates that have made the interpretation of the prognosis of this tumor much more complex than previously assumed. Since 1983, the classification always followed has been the one proposed by Bokhman, who divided endometrial cancer into the following two types: type I or endometrioid and type II or serous [1]. Type I was considered typical of perimenopause, found mainly in obese women, estrogen-dependent, low-grade (G1 or G2), diagnosed earlier and, therefore, at an earlier stage and with a good prognosis [1]. Type II was considered typical of advanced postmenopause, found in not necessarily obese women, non-estrogen-dependent, high-grade (G3), diagnosed later and, therefore, more advanced and with worse prognosis [1]. However, this classification could not fully frame all histological types of endometrial carcinoma and was not shown to be sufficient to appropriately define the prognosis. This is why other pathological features have been considered fundamental for prognoses, such as the depth of myometrial invasion, invasion of the cervical stroma, intra-abdominal disease, lymphovascular space invasion (LVSI), and the presence of nodal metastases [2,3,4].

In 2013, The Cancer Genome Atlas (TCGA) Research Network proposed a new classification of endometrial cancer divided into four molecular groups after genomic, transcriptomic, and proteomic analysis of nearly 400 cases [5]. The four groups were defined as follows: POLE ultramutated, microsatellite instability (MSI) hypermutated, somatic copy number alterations (SCNAs) copy-number low (endometrioid), and SCNAs copy-number high (serous-like) [5]. The POLE ultramutated group, which accounted for 7% of cases, had the best prognosis, and the copy-number high (serous-like) group, which accounted for 26% of cases, had the worst prognosis [5,6]. The other two groups, hypermutated MSI (28%) and copy-number low (endometrioid) (39%), had an intermediate prognosis [5,6]. However, the new classification proposed by TCGA is difficult to apply in the clinical practice because it is too expensive and requires the use of fresh or frozen tumor tissue [5,6].

Since 2015, other research groups have, therefore, tried to make the molecular classification proposed by TCGA more applicable to clinical practice. McConechy et al. [7] demonstrated that the identification of DNA mismatch repair (MMR) deficiencies by immunohistochemistry could effectively replace MSI research in endometrial cancer without having to resort to expensive genomic methodology. Talhouk et al. [8] and Stelloo et al. [9] used p53 immunohistochemistry as a surrogate for copy-number status. Immunohistochemistry was applicable to formalin-fixed paraffin-embedded samples and, therefore, more clinically applicable than genomic and molecular analyses. However, a reliable surrogate of POLE sequencing has not been identified [10,11,12].

Therefore, confirmation and validation studies led to the definition of four types of endometrial carcinoma distinguishable based on clinical, immunohistochemical, and molecular characteristics [10,11,12]. The POLE-mutated type corresponds to the POLE ultramutated group of TCGA and has an excellent prognosis and no relapse. The nonspecific molecular profile (NSMP) or p53 wild-type corresponds to the SCNA copy-number low (endometrioid) group of TCGA and has an intermediate prognosis. The MMR-deficient type corresponds to the hypermutated MSI group of TCGA and has an intermediate-severe prognosis. The p53-abnormal type corresponds to the SCNA copy-number high (serous-like) group of TCGA and has the worst prognosis of all types [10,11,12]. The POLE-mutated type represents between 6 and 9% of cases depending on the studies. The MMR-deficient type represents between 26 and 29% of cases, and the NSMP or p53 wild-type represents between 45% and 59% of cases. The p53-abnormal type represents between 9 and 18% of cases [8,9,10,11,12].

In 2020, the European Society of Gynecological Oncology (ESGO), the European Society for Radiotherapy and Oncology (ESTRO), and the European Society of Pathology (ESP) took note of the large amount of data produced in a short time in an attempt to better classify endometrial cancer from an anatomopathological and molecular point of view, and decided to update the guidelines previously published in 2015 after the conclusion of the work of an evidence-based and multidisciplinary consensus conference involving ESGO, ESTRO, and the European Society for Medical Oncology (ESMO) [13,14]. In these last guidelines, for the first time, joint scientific societies have officially integrated the results of the POLE molecular test and p53 and MMR immunohistochemistry with the stage, grade, and histological type of endometrial carcinoma, as well as with the presence of LVSI in defining risk categories for endometrial cancer [14].

Despite the enormous work carried out in the last decade, many points still remain to be explored. The NSMP or p53 wild-type subgroup of endometrial cancer can be found in 45–59% of endometrial cancer cases and has been defined as having an intermediate prognosis [5,6,7,8,9,10,11,12]. At the same time, the NSMP or p53 wild-type subgroup was associated with several endometrial carcinoma histotypes, such as low-grade endometrioid, high-grade endometrioid, clear cell, undifferentiated or dedifferentiated, neuroendocrine, and carcinosarcoma. However, it accounts for as many as 64% of low-grade endometrioid cancers and 14% of carcinosarcomas [15]. Moreover, some authors have stressed that histotypes of endometrial cancer play a fundamental prognostic role regardless of the molecular group to which they belong because nonendometrioid carcinomas still have a worse prognosis [16]. Above all, the prognosis of the NSMP subgroup seems to depend greatly on the tumor histopathology and the grade of the tumor. The NSMP subgroup is associated with an intermediate prognosis in high-grade endometrioid endometrial cancer and a severe prognosis in nonendometrioid endometrial cancer [17,18]. The NSMP group does not seem to have a well-defined prognostic significance because it does not possess any specific molecular indicator. It could perhaps be further subdivided based on evaluating other molecular alterations such as CTNNB1 mutations and immunohistochemical analysis of altered expression of other proteins such as L1CAM expression [10,19].

It should also be considered that a significant number of endometrial cancers (3–6%) have traits belonging to more than one of the four groups codified by TCGA, so much so that they are defined as “multiple-classifier” [8,9,10,11,12]. The prognosis of some “multiple classifier”, such as cancers with MMR-deficient/p53-abnormal and POLE-mutated/p53-abnormal double classifier, was reported to be significantly different compared to the prognosis of the single classifier p53-abnormal [20].

Although low-grade stage I endometrioid endometrial carcinomas usually have an excellent 5-year prognosis, a small but significant subset of these low-risk cancers can relapse and even die [21,22,23]. It has been hypothesized that this subset of early-stage and low-grade tumors considered low-risk but more prone to recurrence could be identified by identifying CTNNB1 mutations at the molecular level corresponding to the immunohistochemical evaluation of nuclear expression of β-catenin [21,22,23]. However, there is insufficient evidence to accept the use of β-catenin immunohistochemistry for further prognostic classification of endometrial cancer. Some authors have failed to confirm the presence of CTNNB1 mutations in women with low-grade stage I endometrioid endometrial carcinoma that has recurred [15,24].

The search continues for other putative immunohistochemical prognostic markers of relapse-risk in the population of women with low-risk/low-grade endometrioid endometrial cancer. Among the candidate molecules for the role of risk markers in this population, the one on which we have focused our interest in this review is human epididymis protein 4 (HE4).

## 2. HE4

Human epididymis protein 4 (HE4), also known as epididymal secretory protein 4, is a glycoprotein encoded by the Whey-Acidic Four-Disulfide Core domain protein 2 (WFDC2) gene [25]. The genomic sequence coding for the WFDC family of proteins is present at the level of chromosome 20, in the 20q13 region, is 678 kb long and includes 14 coding genes [25]. HE4 is a secretory protein originally identified in the distal human epididymis and shows significant structural similarity to proteinase inhibitors [26,27]. Later, it was shown that the protein is also expressed by the vas deferens, the respiratory epithelium, the salivary glands, the distal tubule of the kidney, and the normal glandular epithelium of the female genital system but not by the superficial ovarian tissue [28]. The genomic sequence encoding the WFDC family of proteins was amplified in various pathological situations, such as carcinoma of the colon, stomach, pancreas, oral cavity, breast, endometrium, and ovary. Therefore, the role of the protein in carcinogenesis and tumor progression has been hypothesized, more precisely, in the migration and adhesion of ovarian cancer cells [28,29,30]. Increased tissue expression of HE4 was then demonstrated in a series of malignancies, particularly of gynecological and pulmonary origin [28,31,32].

Over the years, HE4 has acquired increasing clinical relevance thanks to its ability to differentiate benign adnexal masses from ovarian cancer. Moore’s group in 2012 showed that serum HE4 is less frequently elevated in benign pathologies than CA125 (8% vs. 29%), with better specificity, especially in premenopausal women [33]. In healthy women, serum HE4 levels vary up to 150 pmol/L. This wide range is related to the fact that HE4 values are influenced by age [34], BMI, smoking habits, and respiratory diseases, such as asthma and chronic obstructive pulmonary disease (COPD) [35], as well as the presence of renal failure, which is the main cause of false-positives [36]. Serum levels of HE4 do not appear to be influenced by the phases of the ovarian cycle or by hormonal therapies, which is why HE4 can be measured at any stage of the menstrual cycle (unlike the CA125 marker) and during the use of oral contraceptives [37]. Finally, the concentrations of HE4 across the different trimesters of pregnancy are unchanged relative to the HE4 levels of nonpregnant individuals [34]. However, the serum levels of HE4 reported in the literature greatly depend on the dosage method used by the various authors. Differences between manual and automated HE4 assays have been reported [35]. Over time, several automated immunometric systems that have become available are not yet compatible with each other [38].

Regarding the use of HE4 as a serum biomarker, Moore et al. have developed an algorithm for the calculation of the “ROMA Risk Score” (risk of ovarian malignancy algorithm), which combines the levels of the two biomarkers HE4 and CA 125 and simultaneously takes into account the patient’s menopausal status [39]. The sensitivity of the ROMA Risk Score was 94% (with a specificity of 75%) in distinguishing benign from malignant ovarian pathologies [39,40]. A recent meta-analysis suggested that the ROMA Risk Score is probably more effective in diagnosing epithelial ovarian carcinoma than HE4 and CA125 taken individually in postmenopausal women. At the same time, HE4 is more effective than CA125 in diagnosing epithelial ovarian carcinoma in premenopausal women [41].

Immunohistochemical studies have shown overexpression of HE4 in 100% of ovarian carcinomas of the endometrioid histotype, 93% of ovarian carcinomas of the serous histotype, and 50% of clear cell carcinomas [31]. Lu et al. demonstrated in ovarian cancer cell cultures that the expression of HE4 is associated with cancer cell adhesion and migration [30]. Furthermore, HE4 suppression markedly inhibited cancer growth [30]. Overexpression of HE4 has also been correlated with the proliferation, invasion, and metastasis of ovarian cancer [42]. Finally, HE4 immunocytochemistry appears to be highly sensitive in identifying cells derived from high-grade serous ovarian carcinomas in malignant ascites [43].

However, different isoforms of HE4 protein exist, and different anti-HE4 antibodies recognize different HE4 epitopes [44,45]. In 2002, Bingle et al. [44] described the complex nature of the HE4 gene. The gene undergoes splicing, and HE4 protein exists as five isoforms. The full length HE4 protein contains two whey acidic protein (WAP) domains whereas the other protein isoforms contain only one WAP domain. Two isoforms (HE4-V1 and HE4-V4) contain a N-terminal WAP domain and two (HE4-V2 and HE4-V3) contain a C-terminal WAP domain [44]. The existence of five isoforms of HE4 protein suggested the need to use multiple different antibodies to identify these isoforms both in biological fluids and in healthy or tumor tissues. In 2019, Hellstrom et al. [45] published an interesting study describing the results obtained by using four different anti-HE4 mouse antibodies (mAbs) to characterize the different isoforms of the HE4 protein in ovarian cancer cells and in the blood of women affected by these neoplasms. Two mAbs recognized epitopes on the C terminal and two mAbs recognized epitopes on the N-terminal. The authors determined HE4 binding affinity to the four mAbs and performed sandwich assays for detection of HE4 epitopes. They also performed immunohistochemical evaluation of tumor sections and measured HE4 in sera from women with ovarian carcinoma and in culture supernatants after establishment of ovarian carcinoma cell lines [45]. They evaluated the binding of the four mAbs to tumor sections of 19 ovarian carcinomas and were able to show that one mAb bound to 18 of 19 sections and the other three mAbs bound to 14, 7, and 4 sections out of 19, respectively. Sera from only 12 of the 19 patients had HE4 levels higher than the standard 150 pM cutoff. Cultured cells from ovarian carcinoma lines which expressed high levels of HE4 at immunohistochemistry also secreted the HE4 molecule into the supernatant. Cells which expressed low levels of HE4 at immunohistochemistry did not secrete HE4 into the supernatant. The authors concluded that different anti-HE4 antibodies bind independently in formalin-fixed sections of ovarian carcinomas, identifying subpopulations of ovarian carcinoma cells [45]. Moreover, the overexpression of a specific epitope recognized by a specific anti-HE4 antibody was associated with platinum resistance of ovarian carcinoma cells [45].

## 3. Serum HE4 and Endometrial Cancer

The first evidence suggesting that serum HE4 could be more useful as a tumor biomarker in women with endometrial endometrioid adenocarcinoma than serum CA125 dates back to 2008 [46]. Maintaining a specificity of 95%, the sensitivity of serum HE4 in differentiating women with endometrioid carcinoma of the endometrium at any stage from healthy women was 45.5%, significantly higher than that of serum CA125, which was 24.6% [46].

Since then, several authors have continued to verify the usefulness of serum HE4 as a marker to be used preoperatively in women with endometrial cancer to predict the risk of lymph node metastases [47,48,49]. In 2011, Moore et al. found a high specificity associated with a high sensitivity of serum HE4 in preoperatively distinguishing stage IA from stage IB, i.e., the invasion of less or more than 50% of the myometrium by the carcinoma in 96 women with carcinoma of the endometrium [47]. In 2012, Kalogera et al. verified the high specificity and sensitivity of serum HE4 in predicting myometrial carcinoma invasion and the maximum size of the carcinoma itself in another 75 women [48]. In 2014, Brennan et al. confirmed the previous observations by conducting a similar study in a group of 373 patients identified in a case–control study conducted in Australia between 2005 and 2007 [49]. Elevated serum HE4 levels were significantly associated with reduced recurrence-free survival. They reduced overall survival in the entire population of women with endometrial cancer and in the subpopulation of women with endometrioid cancer of the endometrium and ultimately in the subgroup of women with low-grade (G1/2) endometrioid endometrial cancer [49].

The large amount of data progressively produced by the various researchers was analyzed by various meta-analyses that took place over time from 2014 to 2020 [50,51,52,53]. A meta-analysis published in 2014 focused on the diagnostic accuracy of serum HE4, included six articles and identified high specificity as a strength of the marker [50]. A meta-analysis published in 2018 that included 23 articles also focused on the diagnostic accuracy of serum HE4. It concluded that diagnostic accuracy was higher when the control group consisted of patients with benign diseases than when the control group consisted of healthy women [51]. Different HE4 immunoassays were used in the different studies included in the meta-analysis, which makes the obtained results difficult to interpret [51]. Most of the studies used enzyme immunoassay (EIA), some studies used chemiluminescent microparticle immunoassay (CMIA), and few studies used electrochemiluminescence immunoassay (ECLIA) [51]. A meta-analysis published in 2019 compared the diagnostic accuracy of serum HE4 with that of CA125 in distinguishing women with endometrial cancer from women without cancer [52]. This meta-analysis included 12 studies involving 2586 women, of which 1106 had endometrial cancer, and concluded that the diagnostic accuracy of serum HE4 is better in the Caucasian population than in the Chinese population and is still better than that of CA125 [52]. A fourth meta-analysis was published in 2020 [53]. This meta-analysis included 17 studies with a total of 1807 women with endometrial cancer and 1260 women with healthy or benign uterine disease. It concluded that serum HE4 has good specificity in diagnosing endometrial cancer but relatively low sensitivity, with HE4 threshold values chosen by the various authors ranging from 45.5 to 141.5 mmol/L [53].

In 2021, two systematic reviews were published aiming to verify what advantages the serum HE4 dosage alone or in association with that of CA125 could offer in the diagnosis and in the prognosis and the forecast of survival endometrial carcinoma [54,55]. Degez et al. [54] reported that the sensitivity of HE4 in the diagnosis of endometrial cancer ranged from 44 to 91% for a specificity of 65 to 100%. Serum HE4 levels were related to prognostic factors such as the extent of invasion of myometrium, grade, and stage of carcinoma and presence of lymph node metastases. Behrouzi et al. [55] summarized the performance of serum HE4 as a diagnostic, prognostic, and predictive marker for relapse of endometrial cancer or response to progestogen therapy, especially in women not eligible for classic surgical management through hysterectomy. Serum HE4 appears to be a promising biomarker. However, its clinical use requires considering the limits of confounding variables like age, BMI, smoking habit, respiratory diseases, and the presence of renal failure, which may cause false positives [34,35,36,55]. Furthermore, the threshold levels of serum HE4 chosen by various authors to define the risk of the presence of carcinoma are at least disparate [55]. When we add to this the fact that different authors have used different HE4 immunoassays that have not yet been harmonized, it is easy to understand how difficult it is to homogenize the data reported in the literature to establish a routine clinical use protocol [35,38]. A retrospective observational Italian multicentric study recently demonstrated that different HE4 analytical platforms based on different immunosensors have fixed and/or proportional systematic analytical bias [56]. To identify the ideal threshold beyond which the diagnosis of carcinoma is highly probable, it would, therefore, be necessary to first align the data obtained with different analytical tools using a common reference calibration curve [56].

## 4. Literature Review Aim and Search Strategy

In 2013, HE4 was demonstrated to promote tumor growth in endometrial cancer cell lines and in a mouse xenograft model [57]. Two different endometrial cancer cell lines were transfected with HE4 DNA to achieve clones with HE4 overexpression. Compared to control clones, clones with HE4 overexpression showed increased proliferation, increased matrigel invasion activity, and increased colony formation in soft agar [57]. Clones with HE4 overexpression and control clones were injected into severe combined immunodeficient mice. Tumors formed by clones with HE4 overexpression showed accelerated growth compared to those formed by control clones. The final weight of tumors formed by clones with HE4 overexpression was significantly heavier than that of tumors by control clones [57].

The purpose of this review was to verify how many authors have thus far focused their research on the tissue expression of HE4 in patients with endometrial cancer and its significance with respect to various clinical survival parameters. This is an attempt to identify other immunohistochemical prognostic markers in women with endometrial cancer.

All articles citing the expression of HE4 in the tissue of pathologically diagnosed endometrial cancer or atypical endometrial hyperplasia were independently identified by three authors (V.M., M.L.F., and M.P.) who conducted a search limited to text words on the following three English databases: PubMed, Web of Science, and Scopus. The following keywords were used: [“endometrial cancer” OR “endometrial carcinoma” OR “endometrial tumor” OR “uterine cancer” OR “endometrial atypical hyperplasia” OR “endometrial hyperplasia” OR “atypical hyperplasia”] AND [“HE4” OR “human epididymis protein 4” OR “human epididymis secretory protein 4” OR” human epididymis-specific protein 4” OR “WFDC2”] AND [“immunohistochemistry” OR “expression” OR “overexpression”]. Each database was searched from its initial inclusion date to December 2021. To identify all possible relevant studies, regardless of study year, publication status or type of article were applied to the search. Only non-English-language articles were excluded unless they had an English abstract. All relevant studies were identified by the same three authors (V.M., M.L.F., and M.P.). Additionally, a manual search of reference lists of the retrieved publications was also completed until no additional articles could be identified. The authors did not check abstract books of recent international conferences.

## 5. Search Results

A total of 13 studies relevant to the search scope were found [28,31,58,59,60,61,62,63,64,65,66,67,68]. Of these, 12 are in English [28,31,58,59,60,61,62,63,64,65,66,67]. One is in Chinese but with the abstract in English [68].

### 5.1. HE4 Tissue Protein Expression

As reported in Table 1, HE4 tissue protein expression was measured by immunohistochemistry using either a tissue microarray or tissue slides in 11 studies [28,31,59,61,62,63,64,65,66,67,68]. Overall, in the 11 studies, HE4 tissue protein expression was measured in 109 normal endometrial specimens, 281 endometrial hyperplasia specimens, and 524 endometrial carcinoma specimens [28,31,59,61,62,63,64,65,66,67,68]. Several anti-HE4 antibodies, such as rabbit polyclonal antibody, rabbit monoclonal antibody, or clone 12A2 monoclonal IgG1 antibody, have been used in various studies [28,31,59,61,62,63,64,65,66,67,68]. It should also be considered that in the different studies, rabbit polyclonal antibodies were provided by different laboratories, such as Covance (Dedham, MA, USA) or Abcam (Cambridge, UK), as well as monoclonal antibodies provided by Fujirebio Diagnostics, Inc. (Malvern, PA, USA) or Abcam (Cambridge, UK) [59,61,62,64,66]. Furthermore, the antibody dilutions ranged from 1:20 to 1:400 (Table 1) [28,59,61,62].

In 2005, Drapkin et al. [31] performed a tissue immunohistochemical assessment of the presence of the HE4 protein together with a measurement of tissue RNA expression for HE4 in both normal and pathological anatomical samples, both ovarian and nonovarian. For the first time, immunohistochemical localization of HE4 protein was positive in 4 out of 4 normal endometrium specimens and in 3 out of 4 endometrial carcinoma specimens (Table 1) [31].

In 2006, Galgano et al. [28] evaluated HE4 immunoreactivity in tissue microarrays of 448 ovarian and nonovarian tumors, including 16 endometrioid-type endometrial carcinomas. Cytoplasmic staining was graded for intensity and percentage of positive cells [28]. The intensity and percentage grades were multiplied to obtain an H-score. Immunohistochemical staining for HE4 was present in 14 of 16 endometrioid-type endometrial carcinomas and 11 had a strong H-score (Table 1) [28].

In 2011, Bignotti et al. [59] investigated both HE4 tissue protein expression and HE4 tissue mRNA expression in a large number of endometrial cancer cases compared to normal endometrial specimens both in the proliferative and secretory phases of the cycle (Table 1 and Table 2). HE4 tissue protein expression was evaluated in 153 endometrial carcinoma cases, including 100 low-grade (G1 or G2) cases and 53 high-grade (G3) cases, and in 33 healthy endometrial samples, 16 in the proliferative phase and 17 in the secretory phase of the cycle [59]. Furthermore, 34 of the 153 endometrial carcinoma cases were nonendometrioid [59]. Cytoplasmic staining was graded for intensity and percentage of positive cells [59]. The intensity and percentage scores were multiplied to obtain a total score ranging from 0 (negative) to 3 [59]. Healthy endometrial samples preferentially showed a low immunoreactivity score [59]. Among the cases of endometrial carcinoma, low-grade G1/G2 carcinomas were positive in a higher percentage than G3 carcinomas and nonendometrioid carcinomas [59].

Additionally, in 2011, an article in Chinese was published, the abstract of which can be found in Scopus in English [68]. HE4 tissue protein expression was detected by immunohistochemistry in 20 healthy endometrial samples in the proliferative phase of the cycle, in 31 endometrial carcinoma cases and, for the first time, in 19 cases of endometrial hyperplasia [68]. HE4 tissue protein expression was higher in endometrial carcinoma cases than in healthy endometrial samples and endometrial hyperplasia [68]. HE4 tissue protein expression in endometrial carcinoma cases was correlated with the staging [68].

In 2015, the same Chinese research group published two papers [61,62]. In the first study, Deng et al. [61] evaluated the co-expression of HE4 protein and Annexin A2 (ANXA2), a protein significantly increased in malignant tumors, in 84 endometrial cancer samples, 30 cases of atypical endometrial hyperplasia, and 18 healthy endometrial specimens. Staining was graded for intensity and percentage of positive cells [61]. The intensity and percentage scores were multiplied to obtain a total score ranging from – to +++ [61]. Immunohistochemical staining for HE4 was positive in 72 out of 84 (85.7%) endometrial cancer samples, 20 out of 30 (66.7%) cases of atypical endometrial hyperplasia, and 3 out of 18 (16.7%) healthy endometrial samples [61]. Endometrial cancer cases with high staining scores of ANXA2 and HE4 had a shortened overall survival [61].

In the second paper by the same Chinese research group, Li et al. [62] investigated HE4 tissue protein expression by immunohistochemistry in 102 endometrial cancer samples, 30 cases of endometrial atypical hyperplasia, and 20 healthy endometrial specimens. HE4 tissue protein expression in endometrial cancer samples was correlated with staging, and mortality was higher in patients with higher protein expression [62].

In 2020, Celik et al. [63] compared the HE4 protein expression observed in 23 cases of serous carcinoma of the uterus with that observed in 29 cases of serous carcinoma of the ovary. Cytoplasmic staining was graded for intensity and percentage of positive cells [63]. Almost 61% of serous carcinomas of the uterus were negative for the immunostaining of HE4 protein [63].

In 2021, Cuesta-Guardiola et al. [64] published the results of a prospective case–control study conducted in Spain. They recruited 35 cases of endometrial cancer and 34 cases of healthy endometrium. Cytoplasmic staining was graded for intensity and percentage of positive cells [64]. The intensity and percentage scores were multiplied to obtain a total score defined as 0, 1, and 2 [64]. Of the 35 cases of endometrial cancer, 5 had a total score of 0, 8 had a total score of 1, and 22 had a total score of 3 [64].

Additionally, in 2021, El-Hamed et al. [65] published their study on 40 cases of endometrial cancer and 30 cases of endometrial hyperplasia, 15 atypical and 15 nonatypical. Of the 40 cases of endometrial cancer, 8 (20%) had a negative total score, 17 (42.5%) had a weak total score, and 15 (37.5%) had a strong total score [65]. Of the 15 cases of atypical endometrial hyperplasia, 3 (20%) had a negative total score, 9 (60%) had a weak total score, and 3 (20%) had a strong total score [65]. In contrast, nonatypical endometrial hyperplasia had mainly a negative total score, 10 out of 15 (67%), or a weak total score, 5 out of 15 (33%), and no cases of a strong total score [65].

Orbo et al. [66] and Behrouzi et al. [67] focused their attention on HE4 as a tissue marker to predict the response to progestin therapy in endometrial hyperplasia and early-stage endometrial cancer. In 2016, Orbo et al. [66] obtained biopsy material from 141 women with endometrial hyperplasia who participated in a multicenter randomized study aimed at evaluating the results of 6 months of treatment with a levonorgestrel-impregnated intrauterine device (n = 48), with continuous oral medroxyprogesterone acetate (n = 44), or finally with cyclic oral medroxyprogesterone acetate (n = 44). Before the start of treatment, 8 biopsies had low H-scores, 109 had medium H-scores, and 24 had high H-scores [66].

In 2020, Behrouzi et al. [67] obtained biopsy material from 31 women with atypical endometrial hyperplasia and 36 women with endometrioid endometrial adenocarcinomas (32 were G1 and 4 were G2) who were unsuitable for surgery or asked for fertility-sparing therapy and then participated in a prospective study aimed at evaluating the results of 12 months of treatment with a levonorgestrel-impregnated intrauterine device. Before the start of treatment, out of 31 biopsies of atypical endometrial hyperplasia, 12 had low H-scores, 13 had medium H-scores, and 6 had high H-scores [67]. Out of 36 biopsies of endometrioid endometrial adenocarcinoma, 18 had low H-scores, 13 had medium H-scores, and 5 had high H-scores [67].

Taken together, the data obtained by the authors who measured tissue protein expression by immunohistochemistry suggest that HE4 expression is higher in endometrial carcinoma than in normal endometrium and endometrial hyperplasia. The highest rates of positivity are observed in low-grade G1/G2 endometrioid carcinomas and the lowest rates are observed in serous carcinomas. Furthermore, low-grade G1/G2 endometrioid carcinomas show the highest percentage of high immunostaining scores. High staining scores correlate with shortened overall survival [28,31,59,61,62,63,64,65,68].

### 5.2. HE4 Tissue RNA Expression

As reported in Table 2, HE4 tissue RNA expression was measured as mRNA by either oligonucleotide microarray technology or quantitative real-time PCR in 3 studies [58,59,60]. One study investigated HE4 tissue RNA expression and HE4 tissue protein expression [59]. Overall, in the 3 studies HE4 tissue RNA expression was measured in 77 normal endometrial specimens and 103 endometrial carcinomas [58,59,60].

In 2011, Bignotti et al. [59] investigated both HE4 tissue mRNA expression and HE4 tissue protein expression in a large number of endometrial cancer cases compared to normal endometrial specimens both in the proliferative and secretory phases of the cycle (Table 1 and Table 2). HE4 tissue mRNA expression was evaluated in 46 endometrial carcinoma cases, including 26 low-grade (G1 or G2) cases and 20 high-grade (G3) cases, and in 20 healthy endometrial samples, showing that mRNA expression was 2.6 times greater in carcinoma tissues than in healthy endometrium (Table 2) [59].

In 2013, Jiang et al. [60] demonstrated for the first time the differential expression of five HE4 mRNA variants (i.e., V0, V1, V2, V3, and V4) in 16 healthy endometrial samples and 43 endometrial cancer tissues (14 endometrioid G1, 14 endometrioid G3 and 15 papillary serous) to establish an HE4 variant-specific correlation with survival. HE4-V0, HE4-V1, and HE4-V4 were higher in G1 and G3 endometrioid endometrial cancer than in healthy endometrium [60]. HE4-V2 and HE4-V3 were higher in G1 endometrial cancer than in healthy endometrium [60]. All variants except HE4-V0 were higher in papillary serous endometrial cancer than in healthy endometrium [60]. The levels of mRNA variants HE4-V1, HE4-V3, and HE4-V4 were inversely correlated with survival, while mRNA variant HE4-V0 were positively correlated with age in G1 and G3 endometrioid endometrial cancer [60]. However, no correlation was found between the levels of any of the five HE4 mRNA variants and the survival or age of patients with papillary serous endometrial cancer [60].

Taken together, the data obtained by the authors who measured HE4 tissue mRNA expression suggest that HE4 mRNA is detectable in normal endometrium and endometrial cancer. However, in the latter, the mRNA expression is greater [58,59,60]. Furthermore, the levels of mRNA variants HE4-V1, HE4-V3, and HE4-V4 are inversely correlated with survival, but only in endometrioid endometrial cancer [60].

## 6. Discussion

### 6.1. HE4 Detection in Biological Fluids

Many studies have already been conducted by other authors to evaluate the diagnostic and prognostic capabilities of HE4 detection in biological fluids of women with endometrial cancer. In 2018, Abbink et al. [69] evaluated the role of serum HE4 and CA125 as indicators for recurrence during follow-up. These authors performed a retrospective study including 174 patients with endometrial cancer. They measured HE4 and CA125 serum levels by using a chemiluminescent enzyme immunoassay (CLEIA) kit and used the cut-off value of 70 pmol/L for HE4 and 35 U/mL for CA125. Serum levels of HE4 were related to myometrial invasion, tumor grade, stage, and lymph node involvement. Thus, serum HE4 was a prognostic factor for reduced overall and disease-free survival [69]. In addition, serum HE4 was able to detect recurrence during follow-up more than 100 days earlier than clinical confirmation in 75% of the patients [69]. In 2021, Cymbaluk-Płoska et al. [70] evaluated the use of serum CA125 and HE4 as prognostic markers in a prospective study that enrolled 349 patients with advanced or recurrent endometrial cancer. HE4 concentrations were measured using an electrochemiluminescence (ECLIA) immunoassay kit and the cut-off value of 70 pmol/L was used for HE4. Serum levels of HE4 and CA125 were related to myometrial invasion, tumor grade, stage, and lymph node involvement. HE4 was a better prognostic factor than CA125. HE4 levels below the cut-off value of 70 pmol/L correlated with better patients’ overall and disease-free survival. In addition, HE4 values below 186 pmol/L were predictive of the possibility of obtaining optimal cytoreductive surgery in the case of single relapse [70]. In 2021, two reviews were published that wanted to verify what advantages serum HE4 offered in the diagnosis, prognosis, and survival forecast in women with endometrial cancer [54,55]. Degez et al. [54] reported that the sensitivity of serum HE4 in the diagnosis of endometrial cancer ranged from 44% to 91%. Serum HE4 levels were related to prognostic factors such as the extent of myometrial invasion, grade, and stage of carcinoma and presence of lymph node metastases. Behrouzi et al. [55] summarized the performance of serum HE4 as a diagnostic, prognostic, and predictive marker for relapse of endometrial cancer and response to progestogen therapy in women not eligible for surgical management. Serum HE4 appeared to be a promising biomarker. However, its clinical use requires considering the limits of confounding variables such as age, BMI, smoking habit, respiratory diseases, and the presence of renal failure, which may cause false positives [55]. Furthermore, the threshold levels of serum HE4 chosen by various authors to define the risk of the presence of carcinoma were not homogeneous [55].

The primary symptom of endometrial cancer is abnormal vaginal bleeding. However, this symptom is not specific for endometrial cancer. For this reason, recent studies have focused on the possibility of using the detection of HE4 on biological fluids for the triage of women with postmenopausal abnormal uterine bleeding. The purpose was to identify women who were really at risk of having endometrial cancer in order to direct only these women to hysteroscopy and endometrial biopsy and spare the invasive diagnostic approach to the 90% of women who had abnormal uterine bleeding but due to nonmalignant pathologies. In 2020, Ge et al. [71] proposed a new risk prediction model based on a combination of markers used for different diseases to have a good endometrial cancer detection rate in women with abnormal vaginal bleeding. The authors performed a retrospective study including 254 women with abnormal vaginal bleeding. The presence of endometrial carcinoma was histologically proven in 127 women while 96 women had atypical endometrial hyperplasia and 31 had no organic disease [71]. The authors measured preoperative serum concentrations of CA125, HE4, CA199, CA153, AFP, and CEA using an automated analyzer based on chemiluminescent enzyme immunoassay and preoperative concentrations of D-dimer and fibrinogen using an automated coagulation analyzer. They employed receiver operating characteristic (ROC) curves to assess the diagnostic efficacy of different tumor markers and different combinations of markers [71]. The most performing risk index of endometrial cancer (RIEC) was obtained by combining serum levels of D-dimer, fibrinogen, HE4, and CA199. HE4 was the most performing single marker in endometrial cancer screening but RIEC showed the best efficiency compared with every single marker [71].

In 2022, Njoku et al. [72] for the first time focused on the diagnostic accuracy of urine CA125 and HE4 levels for the triage of women with postmenopausal abnormal uterine bleeding. Overall, 153 women participated in the study and they were divided into two cohorts. The discovery cohort included 61 women (30 endometrial cancer cases and 31 controls). The validation cohort included 92 (33 cases and 59 controls) [72]. Urine samples were collected prior to any clinical procedures. Urine CA125 and HE4 levels were measured using chemiluminescent enzyme immunoassays. Urine samples had to be diluted to 1:100 prior to HE4 testing. Urine CA125 predicted endometrial cancer with a sensitivity of 72% while HE4 sensitivity was 32%. The small sample size and the lack of data on the molecular classification of endometrial cancer are the two limitations of this study [72].

In 2021, Degez et al. [73] published the proposal to conduct a non-interventional prospective multicenter study designed to better define the diagnostic possibilities offered by plasma CA125 and HE4 assay in a selected population such as that of women with abnormal uterine bleeding. The authors decided to evaluate the sensitivity of HE4 as a priority. They expect to recruit 100 patients over 12 months in three participating centers in France. Plasma CA125 and HE4 concentrations are expected to be measured in a single reference laboratory using an automated analyzer based on electrochemiluminescence immunoassay [73].

### 6.2. HE4 Tissue Expression

The results of this review indicate that a greater number of authors have studied serum HE4 as a diagnostic and prognostic biomarker of endometrial cancer compared to the number of researchers who have studied tissue expression of HE4 in endometrial cancer. It is also surprising that the various authors have never studied correlations between tissue expression of HE4 and the new molecular classification of endometrial cancer. In fact, in various publications, the comparison has always been sought with the pathogenetic classification of Bokhman [1] or the clinical staging of FIGO [2].

Going into more detail, the percentage of positivity of normal endometrial cells to the immunostaining used to detect the presence of the HE4 protein seems to depend very much on the type of antibody and the type of tissue preparation used by the various authors [28,31,59,61,62,63,64,65,66,67,68]. In fact, in the two publications from the same Chinese research group that used a rabbit polyclonal antibody provided by Abcam (Cambridge, UK) on tissue slides, positivity was 15–17% [61,62]. In contrast, in other publications from research groups that used a rabbit polyclonal antibody provided by Covance (Dedham, MA, USA) and a tissue microarray or a rabbit monoclonal antibody provided by Abcam (Cambridge, UK) on tissue slides, the positivity rate was 91–100% [59,64].

Regarding endometrial carcinoma, the percentage of cells positive for immunostaining used to detect the presence of the HE4 protein seems to be linked to the grade and to the histological type of endometrial cancer. The highest rates of positivity are observed in endometrioid carcinomas (85% on average), and the lowest rates are observed in serous carcinomas (39%) [28,31,59,61,62,63,64,65]. Among endometrioid carcinomas, low-grade G1/G2 carcinomas were positive in a higher percentage (93%) than G3 carcinomas [59]. Furthermore, endometrioid carcinomas showed a higher percentage of strong/high immunostaining scores [28,59]. It also seems to be noted that in the three publications by Chinese researchers, HE4 tissue protein expression in endometrial cancer samples was correlated with the staging [61,62,68]. Mortality was higher in patients with higher protein expression [61,62,68].

Regarding endometrial hyperplasia, in the study that recruited the largest number of cases, the percentage of cells’ positivity to immunostaining used to detect the presence of the HE4 protein was, on average equal to 94% [66]. Furthermore, in the studies that differentiated hyperplasia between atypical and nonatypical hyperplasia, the percentage of immunostaining positivity for the HE4 protein was higher in atypical hyperplasia (60–80%) than in nonatypical hyperplasia (33%) [61,62,65,67]. Surprisingly, the two studies that focused their attention on HE4 as a tissue marker to predict the response to progestin therapy provided conflicting results regarding the possibility of predicting the clinical response to progestogen treatment based on the reduction of HE4 tissue expression during treatment [66,67]. In fact, while Orbo et al. [66] showed a correspondence between the reduction of H-scores and the clinical response after 6 months of treatment, Behrouzi et al. [67] showed that there was no significant difference in the proportion of patients who had a reduced H-score among those who had obtained a clinical response versus those who had not between baseline and 3, 6, or 12 months of treatment. However, it should be considered that the two studies are not comparable due to the different number and characteristics of the patients and the different progestin treatments to which the patients were subjected [66,67].

Different levels of five different variants of HE4 mRNA were measured in normal endometrium, low- or high-grade endometrioid endometrial carcinoma, or serous-papillary endometrial carcinoma [60]. Indeed, the variants V0, V1, and V4 were higher in G1 and G3 endometrioid endometrial cancer than in healthy endometrium. The variants V2 and V3 were higher in G1 endometrial cancer than in healthy endometrium. All variants except V0 were higher in papillary serous endometrial cancer than in healthy endometrium [60]. In addition, variants V1, V3, and V4 were inversely correlated with survival in G1 and G3 endometrioid endometrial cancer. The levels of mRNA variant V0 were always positively correlated with age in endometrioid endometrial cancer. However, no correlation was found between the levels of any of the five HE4 mRNA variants and the survival or age of patients with papillary serous endometrial cancer [60]. It can be deduced that each HE4 mRNA variant could have a different diagnostic or prognostic significance in low- and high-grade endometrioid endometrial carcinoma and nonendometrioid endometrial carcinoma. This underlines the importance of resetting all measurements for serum or tissue HE4 levels with a variant-specific approach, and this would be desirable in the case of research focused on the diagnosis and prognosis of endometrial cancer.

The existence of five different variants of HE4 mRNA and multiple protein isoforms of HE4 was first demonstrated by Bingle et al. [44] in 2002. However, all the methods for detecting the presence of HE4 tissue mRNA expression used up to 2013 and all the methods for detecting the presence of HE4 protein expression used by various researchers even after 2013 have always been based on the structure of what was identified as variant V0 in the study published by Jiang et al. [60] in 2013. It is also conceivable that monoclonal antibodies used in immunohistochemistry after 2013 may identify variants other than V0 even if not clearly identified [64,66,67]. Therefore, it may also be true for immunohistochemistry, what has recently been demonstrated for serum HE4, that is, a discrepancy in HE4 serum levels when comparing different manufacturers’ HE4 assays and different HE4 automated immunometric systems [56].

## 7. Conclusions

HE4 enhances endometrial cancer progression in experimental settings.

Few authors have studied HE4 expression in endometrial cancer tissue. From the data of the few identified studies, it emerges that HE4 expression in neoplastic tissue could give information on prognosis mostly in low-grade endometrioid carcinomas.

Studies should now be conducted using antibodies that selectively identify the HE4 variants most useful for assessing the prognosis of low-grade endometrioid carcinomas.

## Figures and Tables

**Table 1:** Characteristics of 11 studies measuring HE4 tissue protein expression by immunohistochemistry.

Reference	Year	Method (Antibody)	Expression Quantification	Tissues (Positive/Total)		
				Normal Endometrium	Endometrial Hyperplasia	Endometrial Carcinoma
Drapkin et al. [31]	2005	tm (r-poly-Ab)	Not performed	4/4	---	3/4
Galgano et al. [28]	2006	tm (r-poly-Ab)	H-score: negative, weak, strong	NR	---	14/16 (strong 11/16)
Bignotti et al. [59]	2011	tm (r-poly-Ab)	Score: 0, 1, 2, 3	31/33	---	130/153
Yang et al. [68].	2011	NR	target integral optical density (IOD)	NR/20	NR/19	NR/31
Deng et al. [61]	2015	ts (r-poly-Ab)	Score: –, +, ++, +++	3/18	20/30	72/84
Li et al. [62]	2015	ts (r-poly-Ab)	Score: –, +, 2+, 3+	3/20	20/30	88/102
Celik et al. [63]	2020	ts (r-poly-Ab)	H-score: negative, weak, strong	---	---	9/23 (strong 7/23) (only serous carcinoma)
Cuesta-Guardiola et al. [64]	2021	ts (r-mono-Ab)	Total score: 0, 1, 2	34/34	---	30/35
El-Hamed et al. [65]	2021	ts (NR)	Final score: –, +, 2+, 3+	---	Nonatypical 5/15 Atypical 12/15	32/40 (only endometrioid carcinoma)
Orbo et al. [66]	2016	ts (12A2- mono-Ab)	H-score: low, medium, high	---	133/141	---
Behrouzi et al. [67]	2020	ts (12A2- mono-Ab)	H-score: low, medium, high	---	19/31 (Atypical)	18/36 (G1 = 17/32 G2 = 1/4)

tm = tissue microarray; ts = tissue slides; r-poly-Ab = rabbit polyclonal antibody; r-mono-Ab = rabbit monoclonal antibody; 12A2- mono-Ab = clone 12A2 monoclonal IgG1 antibody; – = negative; + = weakly positive; ++ = positive; +++ = strongly positive; NR = not reported.

**Table 2:** Characteristics of three studies measuring HE4 tissue mRNA expression.

Reference	Year	Method	Expression Quantification	Tissues (Median mRNA Expression)		
				Normal Endometrium (NE)	Endometrial Hyperplasia	Endometrial Carcinoma (EC)
Huhtinen et al. [58]	2009	OMT *	not reported	7.67	---	8.61
Bignotti et al. [59]	2011	Quantitative real-time PCR	log-scale	not reported	---	significantly higher compared with NE
Jiang et al. [60]	2013	Quantitative real-time PCR	fold over 36B4 mRNA levels	different HE4 variant expression	---	significantly higher expression compared with NE

* OMT = oligonucleotide microarray technology.
